# Preliminary Results of the Adoption and Application of the Integrated Comprehensive Care Bundle Care Program When Treating Patients with Chronic Obstructive Pulmonary Disease

**DOI:** 10.1155/2017/7049483

**Published:** 2017-08-07

**Authors:** Jason R. Guertin, James M. Bowen, Carolyn Gosse, Gord Blackhouse, Daria J. O'Reilly, Emanuel Baltaga, Gerard Cox, Donna Johnson, Brandi Le Blanc, Jane Loncke, Stewart Pugsley, Ravi Sivakumaran, Laura Wheatley, Kevin Smith, Jean-Eric Tarride

**Affiliations:** ^1^Programs for Assessment of Technology in Health, The Research Institute of St. Joe's Hamilton, St. Joseph's Healthcare Hamilton, Hamilton, ON, Canada; ^2^Department of Health Research Methods, Evidence, and Impact, Faculty of Health Sciences, McMaster University, Hamilton, ON, Canada; ^3^Department of Social and Preventive Medicine, Université Laval, Quebec City, QC, Canada; ^4^Centre de Recherche du CHU de Québec, Université Laval, Axe Santé des Populations et Pratiques Optimales en Santé, Hôpital du St-Sacrement, Quebec City, QC, Canada; ^5^St. Joseph's Healthcare Hamilton, Hamilton, ON, Canada; ^6^Department of Medicine, DeGroote School of Medicine, McMaster University, Hamilton, ON, Canada; ^7^Center for Health Economics and Policy Analysis, McMaster University, Hamilton, ON, Canada

## Abstract

**Background:**

St. Joseph's Health System has implemented an integrated comprehensive care bundle care (ICC) program with the hopes that it would improve patients' care while reducing overall costs. The aim of this analysis was to evaluate the performance of the ICC program within patients admitted with chronic pulmonary obstructive disease (COPD).

**Methods:**

We conducted a retrospective observational cohort study comparing ICC patients to non-ICC patients admitted to St. Joseph's Healthcare Hamilton for COPD being discharged with support services between June 2012 and March 2015, using administrative data. Confounding adjustment was achieved through the use of propensity score matching. Medical resource utilizations during the initial hospitalization and within the 60 days following discharge were compared using regression models.

**Results:**

All 76 patients who entered the ICC program (100.0%) were matched 1 : 1 to 76 eligible non-ICC patients (28.4%). Length of stay (6.47 [7.29] versus 9.55 [10.21] days) and resource intensity weights (1.16 [0.80] versus 1.64 [1.69]) were lower in the ICC group within the initial hospitalization but, while favoring the ICC program, healthcare resource use tended not to differ statistically following discharge.

**Interpretation:**

The ICC program was able to reduce initial medical resource utilization without increasing subsequent medical resource use.

## 1. Introduction

**Figure 1:**
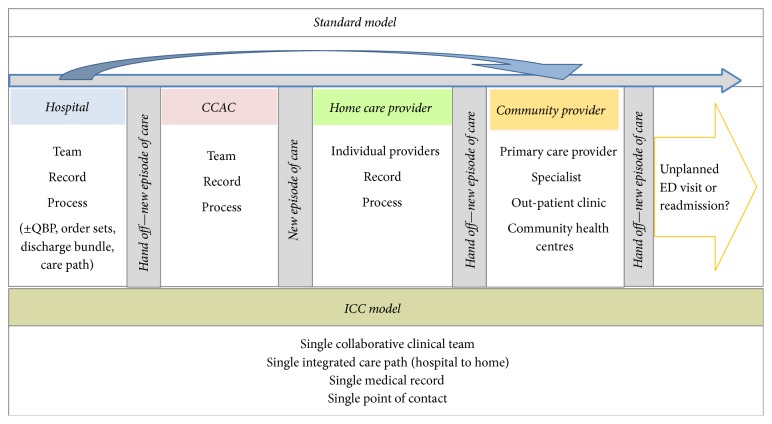
Standard model and ICC model of patient care. CCAC: Community Care Access Centre; ED: emergency department; ICC: integrated comprehensive care; QBP: quality-based procedures.

Healthcare demand and costs are rising in Canada and abroad with the fear that this continuous rise could affect the sustainability of the healthcare systems. Many attempts have been made to curb this increase through the use of multiple hospital/physician/healthcare provider organizations funding models with various degrees of success [1–7]. Most successful attempts tended to encourage coordination of care by multiple healthcare providers which included retroaction to the clinical team in hopes of improving care, aligned incentives for care integration across multiple providers, and optimized resource utilization by the medical teams. In April 2012, St. Joseph's Health System (SJHS) set up a novel patient-centered integrated funding model pilot within the Hamilton (Ontario) region, hereby referred to as the integrated comprehensive care bundle care (ICC) program, designed with these criteria in mind. A detailed description of the ICC program can be found elsewhere [8]. Briefly, the ICC program was designed to integrate care across the hospital and home care continuum, with a single team and care management model that provided homecare support for patients' admitted at St. Joseph's Healthcare Hamilton (SJHH) in collaboration with a lead home care provider, St. Joseph's Home Care (SJHC). Its creation was designed with seven elements in mind: (1) client centered care to empower clients with knowledge, participation, and self-care; (2) integrated care coordinators who would follow clients across the continuum of care; (3) integrated interdisciplinary teams standardizing care pathways spanning hospital and community; (4) use of shared electronic health record which would also serve as a hub for communication; (5) use of simple and available technology to provide flexibility in communication; (6) community-based 24 hours a day, seven days a week access to healthcare; and (7) flexibility in the delivery of care in hopes of continually improving the processes of care. This contrasts with the standard model in which patients' care is in silos and involves multiple additional independent actors including Community Care Access Centres (CCAC) and home care and community providers (Figure 1).

The current ICC model in place at the SJHH is composed of three registered health care professionals who act as the ICC coordinators for all of the clinical streams and are supported by an administrative/research support position. Patients followed within the ICC program have access to the ICC team 24 hours a day, seven days a week through a toll free telephone number. In addition to the ICC personnel, the ICC program leverages an independent version of the SJHC Information System (Procura® ContinuLink® [Victoria, British Columbia]) to provide real-time remote access to client files. Remote access to these files is accomplished by using tablets, laptops, or desktop computers to securely access the information and clinical documentation remotely and provide the option to take digital pictures when required (e.g., wounds).

The ICC program is currently offered to patients in four clinical streams (i.e., thoracic surgery, total joint replacement, congestive heart failure, and chronic obstructive pulmonary disease [COPD]). It was hypothesized that the ICC program could potentially (1) reduce inpatient length of stay (LOS) of the index admission by favoring earlier evaluation for homecare intervention, (2) provide added benefits following discharge, and (3) improve patient and provider experience.

A previous evaluation of the ICC program within the thoracic surgery clinical stream at SJHH showed that, despite having minimal impact within the initial hospitalization, the ICC program was associated with lower proportions of emergency department visits and hospitalization in the postdischarge phase and with a tendency to reduce overall costs [9]. However, it is unknown if these results apply to different populations.

The current manuscript provides the results of our initial evaluation of the performance of the pilot ICC program within the COPD clinical stream for patients admitted to SJHH.

## 2. Methods

### 2.1. Design and Setting

We conducted a comparative retrospective cohort study using data from the Canadian Institute for Health Information (CIHI) Discharge Abstract Database (DAD) linked to the SJHC dataset maintained in Procura ContinuLink.

### 2.2. Study Population

We used CIHI Case Mix Group (CMG) classifications to identify all patients admitted at SJHH for COPD between June 1, 2012, and March 31, 2015. All eligible patients (i.e., community dwelling patients with a hospital admission driven by COPD, requiring home care supports to transition from hospital to home that were not currently receiving care by CCAC, not on a wait list for long-term care or a day program) were approached by the ICC coordinator on the first day of their admission (or on the day of COPD diagnosis for incident COPD cases) for enrollment into the ICC program. Patients in this group who accepted enrollment and were subsequently discharged home formed the ICC group.

**Figure 2:**
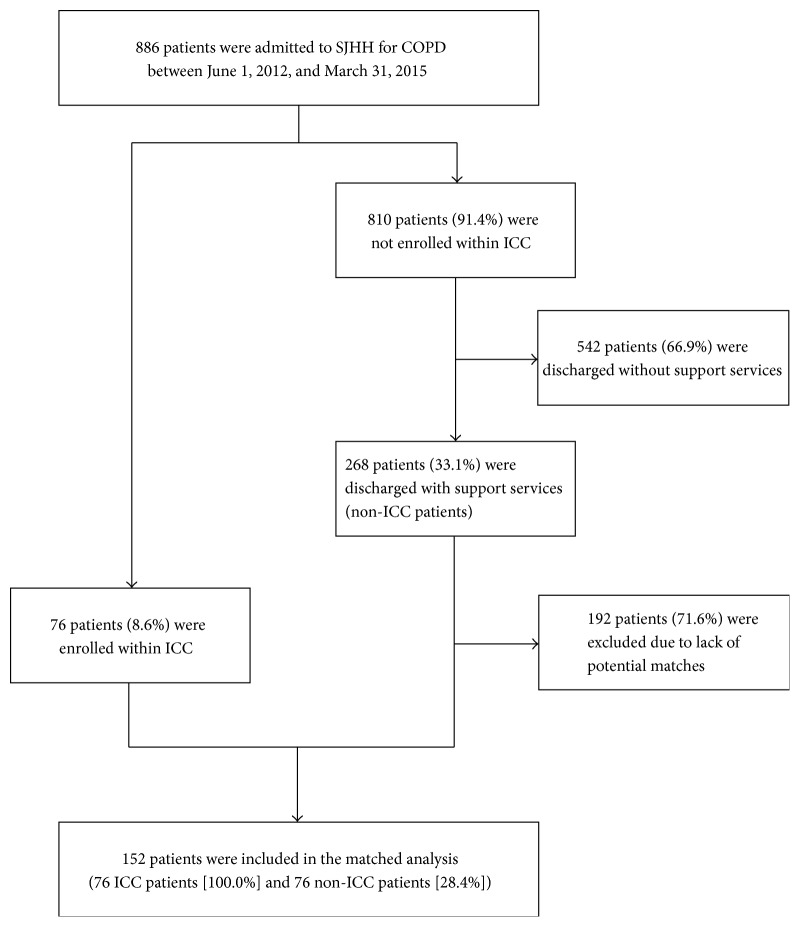
Patient flow-chart of patients admitted for chronic obstructive pulmonary disease at St. Joseph's Healthcare Hamilton. COPD: chronic obstructive pulmonary disease; ICC: integrated comprehensive care; SJHH: St. Joseph's Healthcare Hamilton.

Concurrent comparator patients who were admitted to SJHH for COPD and discharged with standard support services during the study follow-up period were eligible to act as comparator patients (hereby defined as the non-ICC group) (Figure 2). In the event that a patient in the non-ICC group had multiple eligible admissions during the examined time-frame, this patient's index admission was randomly selected among all eligible admissions.

### 2.3. Outcomes

The primary outcome of this study was the in-hospital LOS for the index admission. Secondary outcomes included other in-hospital medical resource utilization rates and included the resource intensity weights (RIW) (i.e., a standard index used by CIHI to reflect intensity of care associated with each hospital admission) [10] of the index admission, the proportion of atypical admissions (defined as admissions in which a patient either has died, was transferred in or out of the hospital, or experienced a long LOS) [11] during the index admission, the number of hospital readmissions, and the total number of days admitted up to 60 days following discharge from index admission. Although 60-day follow-up was considered to be a relevant time-horizon for this study, it could potentially downsize the total number of days patients were admitted during follow-up. As such, this outcome was evaluated using two approaches: (1) a truncated approach where patients' LOS was truncated at the end of the examined time-window and (2) a nontruncated approach where if a patient remained admitted beyond the examined time-window (i.e., 30 or 60 days), the full LOS would be counted (e.g., if a patient was admitted for 5 days on the 29th day of 30-day follow-up, this patient would be assigned a 1-day LOS in the truncated approach and a 5-day LOS in the nontruncated approach).

### 2.4. Statistical Analyses

Discrete data are presented in absolute (number) and relative (percentage) values and continuous data are presented as mean (standard deviation [SD]) or as average rate of event (95% confidence intervals [CI]) per 100 persons; rates were defined per 100 persons per month or per two months in function of the examined follow-up period (i.e., 30 or 60 days). Baseline sociodemographics of all patients included within the ICC and non-ICC group were assessed on the index admission's admission date as well as the numbers of time they were admitted in the 60 days prior to their index admission.

We used propensity score (PS) matching to control for measured baseline confounding between the two groups [12]. Patients' PS were estimated using a multivariate logistic regression PS model which included the following covariates: age, sex, fiscal quarter at the time of patients' discharge from the index admission (fiscal years in Canada go from April 1 to March 31, as such fiscal quarter 1: April to June; fiscal quarter 2: July to September; fiscal quarter 3: October to December; fiscal quarter 4: January to March), and the number of admissions (0, 1 or ≥2) in the 60 days prior to the index admission. Following the selection of the PS model, PS were assessed for all patients included within the ICC and non-ICC groups and matching was conducted using a greedy nearest neighbor 1 : 1 matching algorithm. Matching occurred if the difference in the logit of the PS between nearest neighbors was within 0.2 times the SD of the cohort's PS [13]. Patients selected by the matching algorithm formed the matched population.

Between group balance in terms of the baseline characteristics within the unmatched and matched populations was assessed using absolute standardized differences (ASDD); ASDD > 0.10 were assumed to indicate lack of balance between groups [14, 15]. Proportion of atypical index admission and proportion of patients readmitted within 60 days following discharge of the index admission between the ICC and non-ICC groups were compared using chi-square or Fisher's exact test, when appropriate. Between group differences in terms of LOS for the index admission were compared using negative binomial regressions [16–18] whereas between group differences in the index admission's RIW were compared using generalized linear model with log link and gamma family function [17, 19]. Differences in the rate of hospital readmissions and of in-hospital LOS during follow-up between the ICC and non-ICC patients were compared using zero-inflated negative binomial (ZINB) regressions [17, 19]. These types of model are used when there are a large proportion of 0s in the data (e.g., 135 [88.8%] of 152 matched patients were not hospitalized at 30 days). Rather than using a single distribution to model the count data (e.g., negative binomial distribution), ZINB models use two distributions to account for the excess zeros by implicitly modeling two populations. The first part of the model assumes that some individuals will never be hospitalized using a binary link function (e.g., logit function) while the second part of the model assumes that other individuals can or cannot be hospitalized following a negative binomial distribution. Due to its two-part structure, the ZINB models generate two sets of parameters, one for each part of the model (i.e., zero-inflated part and negative binomial part). All analyses were conducted using the SAS 9.3 Program (Cary, North Carolina). We interpreted 2-tailed *P* values < 0.05 as significant.

## 3. Results

### 3.1. Study Population

A patient flow-chart is shown in Figure 2. Seventy-six patients admitted to SJHH for COPD and discharged home between June 10, 2012, and February 16, 2015, were enrolled within the ICC COPD clinical stream. Aside from these 76 patients, a total of 810 patients have been admitted to SJHH for COPD between June 1, 2012, and March 31, 2015. Of these 810 patients, a total 268 patients (33.1%) admitted to SJHH for COPD and discharged with support services between June 1, 2012, and March 31, 2015, composed the non-ICC group.

### 3.2. Unmatched Analyses

Baseline comparison of the ICC and non-ICC group is shown in Table 1. Although patients in the ICC and non-ICC groups tended to be similar regarding most of the examined baseline characteristics, patients included within ICC group tended to have been admitted to a hospital more frequently in the 60 days prior to the index admission than patients within the non-ICC group (an average of 0.49 [0.72] versus 0.35 [0.66] admission per patient [ASDD = 0.20]), thus justifying the need to adjust for these differences.

### 3.3. Propensity Score Adjusted Analyses

Matched non-ICC comparators were found for all 76 patients in the ICC group. PS matching attained balance on all examined baseline characteristics (Table 2). Outcomes within the ICC group and their matched non-ICC comparators are provided within Table 3 (beta-estimates of the regression models are provided in Table 4). Results indicated that patients within the ICC group had shorter index LOS (6.47 [7.29] versus 9.55 [10.21] days) and used less RIW (1.16 [0.80] versus 1.64 [1.69]) than their matched comparators. In the follow-up period, results failed to indicate any statistically significant difference in terms of readmission rates after discharge at 30-day follow-up (11.84 [4.41; 19.27] versus 13.16 [3.76; 22.56] readmissions per 100 persons) or at 60-day follow-up (23.68 [11.96; 35.41] versus 25.00 [12.55; 37.45] readmissions per 100 persons). Despite this lack of difference in terms of readmission rates, results did indicate that the ICC group had less in-hospital LOS at 30 days after discharge when analyzed under the nontruncated approach (122.37 [31.68; 213.05] versus 536.84 [0.00; 1,189.14] days per 100 patients).

## 4. Interpretation

### 4.1. Main Findings

Evaluation of the ICC program is fundamental to adequately assess its value to patients and the healthcare system [20]. Our study represents the first evaluation of the application of the ICC program to a chronic disease population. Our results indicate that the LOS and RIW within the index admission were lower within the ICC group than within the non-ICC group (Table 3). Such results could raise concerns of the presence of selection bias (i.e., that the ICC coordinators favored less severe cases). However, seeing as the baseline comparisons within the unmatched population indicated that ICC patient utilized more resources prior to baseline (Table 1), such concerns seem unwarranted. With respect to follow-up outcomes, numerical differences in favor of ICC were observed for the 30- and 60-day readmission rates and LOS per 100 persons although most of these results did not attain statistical significance (Table 3). This could be partially explained by the relatively small sample size available for analyses.

### 4.2. Relationship to Other Studies

This is the second pilot evaluation of the performance of the ICC program within SJHH. Though none of the results regarding the ICC program in either stream would indicate worse outcomes with the ICC program, added benefits provided by the ICC program seem to differ between streams. For example, within the COPD stream, benefits with the ICC program were generally observed within the index hospitalization (Table 3), whereas benefits provided within the thoracic surgery stream tended to be in the postdischarge phase [9]. Continued assessment of the performance of the ICC program within these two streams is required to better comprehend if these results reflect true differences regarding the performance of the ICC program at SJHH (i.e., that ICC program provides benefits solely within the index hospitalization within the COPD stream and only within the postdischarge phase within the thoracic surgery stream) or if both studies lacked statistical power to detect a statistically significant result in both phases. Regardless of the case, seeing as the results may differ between the two previously examined streams, evaluation of the two streams which have yet to be evaluated (i.e., total joint replacement and congestive heart failure) is warranted as the performance of the ICC program within these two streams may also differ.

### 4.3. Implications for Practice

Despite these positive results, concerns regarding the fact that both currently available evaluations of the ICC program have been conducted within a research university hospital center and that the results we show may not be reproducible within community hospitals could be raised. It is important to note that the ICC program was later expanded in September 2013 to another SJHS hospital, St. Mary's General Hospital, a community hospital center in Kitchener-Waterloo, Ontario [21]. Although preliminary, initial positive results observed within the SJHS also seem to be observed within this nontertiary setting (full evaluation of this second program is currently underway). These positive and promising results partly support the recent decision of the Ministry of Health and Long-Term Care of Ontario to expand the ICC (for both COPD and CHF patients) program across the Hamilton Niagara Haldimand Brant Local Health Integration Network [22, 23].

### 4.4. Limitations

Our study has several limitations. First, we had access to limited patients' baseline characteristics as well as a limited sample size. Although we used PS matching to further select balanced comparator groups, we may not exclude the risk that we did not fully adjust for measured confounding (e.g., adjusting for seasonality of the index admission would not be able to adjust for acute temporal variability in service-related confounding) nor can we fully exclude the risk of confounding due to unmeasured confounders (e.g., patients' smoking status which was not available within the in-hospital database) [12, 24]. Second, we only had access to the CIHI DAD to conduct this evaluation. Future work evaluating the ICC program will require access to additional CIHI databases, such as the National Ambulatory Care Reporting System, Ontario Case Costing Initiative databases as well as the Client Health Related Information System, and the Registered Persons database, and to be able to link patients' data across these databases to fully examine the ICC program's impact on patients' morbidity and mortality as well as its short- and long-term economic implications. Fourth, our results also highlight the fact that only a limited subset of patients admitted to SJHH for COPD during the study's follow-up period entered within the ICC program (76 [22.1%] out of 344 patients). This observation may reflect issues with the ICC program's capacity which we were unable to evaluate within the current study. Future work will be required to identify the optimal number of ICC coordinators which will be required to fully manage all potential patients. Finally, we recognize that our analyses could have lacked power to detect a significant difference between groups. However, as this evaluation was conducted in a pilot setting where the primary objective was to assess the feasibility of the ICC program not its performance, we did not conduct a power calculation or a sample size estimation prior to conducting the study and did not expect to detect a significant difference in any of the examined outcomes. Despite these limitations, our results regarding the value of the ICC program remain strong (Table 3).

### 4.5. Recommendations for Future Evaluations

From a broader perspective, this evaluation also depicts issues associated with evaluating an intervention which is offered in a nonrandomized manner. Unlike results observed within a randomized controlled study, any differences, or lack of differences, observed within a nonrandomized study may be due to confounding. To address this potential issue, we had determined a priori that confounding adjustment would be conducted in a stepwise approach. First, the ICC program's performance would be evaluated only in patients who could be eligible for inclusion within the ICC program (i.e., those discharged alive with support services following an admission for COPD) and second, subsequent differences in measured baseline characteristics would be further adjusted thanks to a PS matching approach [25]. Had we not selected an appropriate comparator group (baseline comparison of the 76 ICC patients and of the 810 patients admitted to SJHH for COPD is shown in Table 5), we would not have been able to identify that the ICC program could reduce the initial LOS (6.47 [7.29] days in the ICC versus 7.78 [13.92] days in the non-ICC group) (Table 6).

We recommend that any research team planning to conduct a similar analysis reflect on the following points. Firstly, identification and selection of an optimal comparator group are pivotal for the appropriate evaluation of the program. Secondly, all nonrandomized studies are subject to confounding bias; following the selection of the comparator group, researchers must thoroughly examine all available characteristics to identify the level of balance between both groups. Thirdly, following identification of unbalanced characteristics, comparisons must be conducted using optimal adjustment methods (e.g., PS matching). Finally, as shown within our example, full access to all relevant databases and the ability to link data between these databases must be obtained in order to extensively evaluate all short- and long-term implications of such programs across both inpatient and outpatient healthcare services. In addition, the evaluation of these complex healthcare interventions must move beyond the traditional first-level indicators (e.g., readmission [yes versus no]) to better appreciate their impact across the whole spectrum of care.

### 4.6. Conclusion

In conclusion, based on our preliminary results, implementation of the ICC program to the COPD clinical stream within the SJHH statistically reduced patients' index LOS which should reduce in-hospital costs and showed a potential for a reduction in the number of days patients were hospitalized in the 30 and 60 days after discharge. Additional analyses focusing on patients' outcome following discharge, their perception regarding outpatient care, and a full economic evaluation of the ICC program must be conducted prior to concluding on its full value.

## Figures and Tables

**Table 1:** Baseline characteristics within the unmatched ICC and eligible COPD patients.

	ICC group	Non-ICC comparator group^*∗*^	Absolute standardized differences^†^
Number of patients	*N* = 76	*N* = 268	
Male sex, *n* (%)	38 (50.00)	130 (48.51)	0.03
Age, mean (SD)	73.62 (9.18)	73.66 (10.75)	0.00
*N* per quarter (proxy for seasonality), *n* (%)			
FYQ1	18 (23.68)	57 (21.27)	0.06
FYQ2	17 (22.37)	48 (17.91)	0.11
FYQ3	17 (22.37)	79 (29.48)	0.16
FYQ4	24 (31.58)	84 (31.34)	0.01
Prior admission in the 60 days prior to baseline			
No admission, *n* (%)	47 (61.84)	198 (73.88)	0.26
1 prior admission, *n* (%)	23 (30.26)	51 (19.03)	0.26
2 or more prior admissions, *n* (%)	6 (7.89)	19 (7.09)	0.03
*N* of admissions in the 60 days prior to baseline admission, mean (SD)	0.49 (0.72)	0.35 (0.66)	0.20

FYQ, fiscal year quarter; *N*, number; SD, standard deviation. ^*∗*^The comparator group within this table was composed of all patients who did not enter within the ICC group and who were admitted for COPD to St. Joseph's Healthcare Hamilton and discharged between June 1, 2012, and March 31, 2015. ^†^Absolute standardized differences > 0.10 are generally assumed to indicate lack of balance between groups.

**Table 2:** Baseline characteristics within the matched population.

	ICC group	Matched non-ICC group^*∗*^	Absolute standardized differences^†^
Number of patients	*N* = 76	*N* = 76	
Male sex, *n* (%)	38 (50.00)	38 (50.00)	0.00
Age, mean (SD)	73.62 (9.18)	74.49 (9.90)	0.09
*N* per quarter (proxy for seasonality), *n* (%)			
FYQ1	18 (23.68)	15 (19.74)	0.10
FYQ2	17 (22.37)	18 (23.68)	0.03
FYQ3	17 (22.37)	17 (22.37)	0.00
FYQ4	24 (31.58)	26 (34.21)	0.06
Prior admission in the 60 days prior to baseline			
No admission, *n* (%)	47 (61.84)	47 (61.84)	0.00
1 prior admission, *n* (%)	23 (30.26)	22 (28.95)	0.03
2 or more prior admissions, *n* (%)	6 (7.89)	7 (9.21)	0.05
*N* of admission in the 60 days prior to baseline admission, mean (SD)	0.49 (0.72)	0.49 (0.70)	0.00

FYQ, fiscal year quarter; *N*, number; SD, standard deviation. ^*∗*^The comparator group within this table was composed of eligible non-ICC patients that were propensity score-matched to an ICC patient. Patients were matched on patients' gender, age, the fiscal quarter in which they were discharged from the hospital for a COPD admission, and number of prior admissions in the last 60 days. ^†^Absolute standardized differences > 0.10 are generally assumed to indicate lack of balance between groups.

**Table 3:** Medical resource utilization within the index admission and up to 60-day follow-up within the matched population.

	ICC group	Matched non-ICC comparator group^*∗*^	*P* value	
Number of patients	*N* = 76	*N* = 76		

Length of stay during initial hospitalization (days) (mean (SD); min–max)	6.47 (7.29) [1; 42]	9.55 (10.21) [1; 57]	<0.01	

Resource intensity weight (RIW) during initial hospitalization (mean (SD); min–max)	1.16 (0.80) [0.17; 5.53]	1.64 (1.69) [0.73; 11.63]	<0.01	

Proportion of atypical index admission, *n* (%)	4 (5.26)	4 (5.26)	1.00	

Proportion of patients with at least 1 readmission, *n* (%)				
At 30 days	9 (11.84)	8 (10.53)	0.80	
At 60 days	15 (19.74)	15 (19.74)	1.00	

Average readmission rates per 100 persons (95% CI)				
At 30 days	11.84 (4.41; 19.27)	13.16 (3.76; 22.56)	0.07^‡^	0.99^§^
At 60 days	23.68 (11.96; 35.41)	25.00 (12.55; 37.45)	0.72^‡^	0.76^§^

Average LOS per 100 persons (95% CI)				
Truncated at 30 days	94.74 (27.58; 161.89)	136.84 (27.36; 246.33)	0.11^‡^	0.78^§^
Truncated at 60 days	236.84 (103.71; 369.97)	285.53 (73.10; 497.95)	0.43^‡^	0.80^§^

Average LOS (nontruncated) per 100 persons (95% CI)^†^				
At 30 days	122.37 (31.68; 213.05)	536.84 (0.00; 1189.14)	<0.01^‡^	0.56^§^
At 60 days	260.53 (110.56; 410.49)	609.21 (0.00; 1261.88)	0.07^‡^	0.76^§^

LOS, length of stay; *N*, number; SD, standard deviation. No adjustment beyond the propensity score matching was conducted. Beta-estimates (95% CI) for each statistical comparison are provided within Table 4. ^*∗*^The comparator group within this table was composed of eligible non-ICC patients that were propensity score-matched to an ICC patient. Patients were matched on patients' gender, age, the fiscal quarter in which they were discharged from the hospital for a COPD admission, and number of prior admissions in the last 60 days. ^†^For patients still admitted at the end of the examined time-horizon, the complete LOS was included in the analysis even if it extended beyond the examined time-horizon. ^‡^*P* value within the negative binomial part of the ZINB regression. ^§^*P* value within the zero-inflated part of the ZINB regression.

**Table 4:** Results of the statistical comparisons of the outcomes within the matched population.

	Beta-estimates (95% CI)			*P* value
Length of stay during initial hospitalization	−0.39 (−0.67–−0.11)			<0.01
Resource intensity weight (RIW) during initial hospitalization	−0.34 (−0.53–−0.16)			<0.01
Proportion of atypical index admission^*∗*^	—			1.00
Proportion of patients with at least 1 readmission^*∗*^				
At 30 days	—			0.80
At 60 days	—			1.00
	Negative binomial part	*P* value	Zero-inflated part	*P* value
	Beta-estimates (95% CI)		Beta-estimates (95% CI)	
Average readmission rates per 100 persons				
At 30 days	−1.37 (−2.86–0.12)	0.07	−14.52 (−2324.47–2295.43)	0.99
At 60 days	−0.27 (−1.72–1.18)	0.72	−0.50 (−3.74–2.74)	0.76
Average LOS per 100 persons				
At 30 days	−0.50 (−1.11–0.11)	0.11	−0.15 (−1.16–0.87)	0.78
At 60 days	−0.27 (−0.94–0.40)	0.43	−0.10 (−0.92–0.72)	0.80
Average LOS (nontruncated) per 100 persons				
At 30 days	−1.75 (−3.01–−0.49)	<0.01	−0.31 (−1.38–0.75)	0.56
At 60 days	−0.95 (−1.99–0.08)	0.07	−0.14 (−1.02–0.74)	0.76

^*∗*^ *P* values estimated by chi-square or Fischer's exact test. The comparator group used for these analyses was composed of eligible non-ICC patients that were propensity score-matched to an ICC patient. Patients were matched on patients' gender, age, the fiscal quarter in which they were discharged from the hospital for a COPD admission, and number of prior admissions in the last 60 days.

**Table 5:** Baseline characteristics within the unselected population.

	ICC group	Unselected COPD comparison group^*∗*^	Absolute standardized differences†
Number of patients	*N* = 76	*N* = 810	
Male sex, *n* (%)	38 (50.00)	406 (50.12)	0.00
Age, mean (SD)	73.62 (9.18)	72.17 (11.42)	0.14
*N* per quarter (proxy for seasonality), *n* (%)			
FYQ1	18 (23.68)	161 (19.88)	0.09
FYQ2	17 (22.37)	155 (19.14)	0.08
FYQ3	17 (22.37)	231 (28.52)	0.14
FYQ4	24 (31.58)	263 (32.47)	0.02
Prior admission in the 60 days prior to baseline, *n* (%)			
No admission	47 (61.84)	645 (79.63)	0.40
1 prior admission	23 (30.26)	126 (15.56)	0.36
2 or more prior admissions	6 (7.89)	39 (4.81)	0.13
*N* of admissions in the 60 days prior to baseline admission, mean (SD)	0.49 (0.72)	0.27 (0.60)	0.33

FYQ, fiscal year quarter; *N*, number; SD, standard deviation. ^*∗*^The comparison group within this table is composed of all 810 patients admitted to St. Joseph's Healthcare Hamilton for COPD between June 1, 2012, and March 31, 2015, who did not enter within the ICC group. ^†^Absolute standardized differences > 0.10 are generally assumed to indicate lack of balance between groups.

**Table 6:** Example of the unadjusted analyses using the inappropriate COPD comparator group.

	ICC group	Unselected COPD comparison group^*∗*^	*P* value	
Number of patients	*N* = 76	*N* = 810		

Length of stay during initial hospitalization (days) (mean (SD); min–max)	6.47 (7.29) [1; 42]	7.78 (13.92) [1; 226]	0.13	

Resource intensity weight (RIW) during initial hospitalization (mean (SD); min–max)	1.16 (0.80) [0.17; 5.53]	1.56 (2.09) [0.18; 27.32]	<0.01	

Proportion of atypical index admission, *n* (%)	4 (5.26)	71 (8.77)	0.39	

Proportion of patients with at least 1 readmission, *n* (%)				
At 30 days	9 (11.84)	73 (9.01)	0.42	
At 60 days	15 (19.74)	128 (15.80)	0.37	

Average readmission rates per 100 persons (95% CI)				
At 30 days	11.84 (4.41; 19.27)	9.63 (7.45; 11.81)	0.82^‡^	1.00^§^
At 60 days	23.68 (11.96; 35.41)	18.64 (15.44; 21.85)	0.87^‡^	0.82^§^

Average LOS per 100 persons (95% CI)				
At 30 days	94.74 (27.58; 161.89)	64.81 (44.98; 84.65)	0.80^‡^	0.39^§^
At 60 days	236.84 (103.71; 369.97)	148.89 (110.59; 187.19)	0.43^‡^	0.36^§^

Average LOS (nontruncated) per 100 persons (95% CI)^†^				
At 30 days	122.37 (31.68; 213.05)	149.14 (70.07; 228.20)	0.34^‡^	0.31^§^
At 60 days	260.53 (110.56; 410.49)	212.59 (130.95; 294.23)	0.96^‡^	0.39^§^

LOS, length of stay; *N*, number; SD, standard deviation. Beta-estimates (95% CI) for each statistical comparison are provided in Table 7. ^*∗*^The comparison group within this table is composed of all 810 patients admitted to St. Joseph's Healthcare Hamilton for COPD between June 1, 2012, and March 31, 2015, who did not enter within the ICC group. ^†^For patients still admitted at the end of the examined time-horizon, the complete LOS was included in the analysis even if it extended beyond the examined time-horizon. ^‡^*P* value within the negative binomial part of the ZINB regression. ^§^*P* value within the zero-inflated part of the ZINB regression.

**Table 7:** Results of the statistical comparisons of the outcomes within the inappropriate COPD comparator group.

	Beta-estimates (95% CI)			*P* value
Length of stay during initial hospitalization	−0.18 (−0.42–0.05)			0.13
Resource intensity weight (RIW) during initial hospitalization	−0.29 (−0.46–−0.13)			<0.01
Proportion of atypical index admission^*∗*^	—			0.39
Proportion of patients with at least 1 readmission^*∗*^				
At 30 days	—			0.42
At 60 days	—			0.37
	Negative binomial part		Zero-inflated part	
	Beta-estimates (95% CI)	*P* value	Beta-estimates (95% CI)	*P* value
Average readmission rates per 100 persons				
At 30 days	−0.12 (−1.21–0.96)	0.82	−14.57 (−6017.00–5987.87)	1.00
At 60 days	0.10 (−1.07–1.27)	0.87	−0.34 (−3.19–2.52)	0.82
Average LOS per 100 persons				
At 30 days	0.09 (−0.61–0.78)	0.80	−0.33 (−1.08–0.43)	0.39
At 60 days	0.23 (−0.34–0.81)	0.43	−0.29 (−0.90–0.33)	0.36
Average LOS (nontruncated) per 100 persons				
At 30 days	−0.57 (−1.74–0.60)	0.34	−0.46 (−1.34–0.42)	0.31
At 60 days	−0.02 (−0.81–0.77)	0.96	−0.30 (−0.97–0.37)	0.39

^*∗*^ *P* values estimated by chi-square or Fischer's exact test. The comparison group used in these analyses is composed of all 810 patients admitted to St. Joseph's Healthcare Hamilton for COPD between June 1, 2012, and March 31, 2015, who did not enter within the ICC group.
